# Risk of Chromosomal Abnormalities in Early Spontaneous Abortion after Assisted Reproductive Technology: A Meta-Analysis

**DOI:** 10.1371/journal.pone.0075953

**Published:** 2013-10-10

**Authors:** Jun-Zhen Qin, Li-Hong Pang, Min-Qing Li, Jing Xu, Xing Zhou

**Affiliations:** 1 Department of Obstetrics and Gynecology, First Affiliated Hospital of Guangxi Medical University, Nanning, China; 2 Department of Hepatobiliary Surgery, First Affiliated Hospital of Guangxi Medical University, Nanning, China; 3 Department of Respiratory Medicine, Third People of Guangxi Zhuang Autonomous Region, Nanning, China; University of Nevada School of Medicine, United States of America

## Abstract

**Background:**

Studies on the risk of chromosomal abnormalities in early spontaneous abortion after assisted reproductive technology (ART) are relatively controversial and insufficient. Thus, to obtain a more precise evaluation of the risk of embryonic chromosomal abnormalities in first-trimester miscarriage after ART, we performed a meta-analysis of all available case–control studies relating to the cytogenetic analysis of chromosomal abnormalities in first-trimester miscarriage after ART.

**Methods:**

Literature search in the electronic databases MEDLINE, EMBASE, and Cochrane Central Register of Controlled Trials (CENTRAL) based on the established strategy. Meta-regression, subgroup analysis, and Galbraith plots were conducted to explore the sources of heterogeneity.

**Results:**

A total of 15 studies with 1,896 cases and 1,186 controls relevant to the risk of chromosomal abnormalities in first- trimester miscarriage after ART, and 8 studies with 601 cases and 602 controls evaluating frequency of chromosome anomaly for maternal age≥35 versus <35 were eligible for the meta-analysis. No statistical difference was found in risk of chromosomally abnormal miscarriage compared to natural conception and the different types of ART utilized, whereas the risk of fetal aneuploidy significantly increased with maternal age≥35 (OR 2.88, 95% CI: 1.74–4.77).

**Conclusions:**

ART treatment does not present an increased risk for chromosomal abnormalities occurring in a first trimester miscarriage, but incidence of fetal aneuploidy could increase significantly with advancing maternal age.

## Introduction

Assisted reproductive technology (ART) has been an important therapy method and a basic technique in many infertile couples to have children. Although the ART pregnancy rate of multiple Infertility treatment centers is stable at around 40%, the take home baby rate is still 20–30%, one of the important reasons is the high rate of early spontaneous abortion [1], [2]. First-trimester miscarriage occurs in 10%–15% of all clinical recognized pregnancies, with embryonic chromosomal abnormalities being the most common cause of spontaneous miscarriage, which accounting for approximately 60% of these pregnancy losses [3], [4]. However, the rate of early spontaneous abortion in patients after ART is ranging from 22%–63%. The failure of ART treatment is associated with many factors, genetic defects especially embryonic chromosomal abnormalities, are one of the major causes of spontaneous miscarriage during the first trimester [4], [5].

Cytogenetic analysis of products of conception (POC) is essential to examine the cause of the spontaneous abortion. Multiple cytogenetic analysis have indicated aneuploidy rates of first trimester miscarriages ranging from 50%–80% in various populations. The abnormal karyotypes from of cytogenetic studies include autosomal trisomies, sex chromosome monosomy, triploidy, double trisomies, polyploidies, as well as structural rearrangements. Among these autosomal trisomies are the most common chromosomal abnormalities [6], [7]. Moreover, it has been speculated that the different type of assisted reproductive technologies utilized,which including in vitro fertilization-embryo transfer(IVF-ET)and intracytoplasmic sperm injection(ICSI) and frozen embryo transfer (F-ET)and intrauterine insemination (IUI, may determine the risk of cytogenetically abnormal products of conception [7], [8].

There are adverse factors of advanced maternal age, altered karyotype, multiple assisted reproductive technologies (ART) failure, repeated miscarriages, spermatozoa obtained by Mesa-Tese reported, which lead to an increased risk of embryonic chromosomal abnormalities [9], [10]. Maternal age is probably considered to be the most important factor in pregnancy outcome in ART. For infertility couples, the risks of miscarriage and aneuploidy rate are known to be highly increases with advancing maternal age [11], [12]. Although the pathogenesis of the age effect is not fully understood, it is considered to be contributed to by errors arising at meisois I in the oocyte [13].

A number of studies have been conducted to investigate the pregnancy outcomes after ART, and the relationship between risk of chromosomal abnormalities occurring in first-trimester miscarriage and ART treatment, but their results are somewhat controversial and underpowered. With regards to whether an elevated risk of chromosomal abnormalities resulting in a first trimester miscarriage after ART, to the best of our knowledge, no meta-analyses on this issue have ever appeared. To obtain a more precise evaluation of the association between embryonic chromosomal abnormalities and risk of first-trimester miscarriage after ART, we performed a meta-analysis of all available case–control studies relating the cytogenetic analysis of chromosomal abnormalities to risk of first-trimester miscarriage after ART.

## Methods

### Search strategy

To select eligible studies, a search was performed in the electronic databases MEDLINE, EMBASE, and Cochrane Central Register of Controlled Trials (CENTRAL), using the search strategy depended on various combinations of the keywords “assisted reproductive technology, ART or intracytoplasmic sperm injection, ICSI or in vitro fertilization-embryo transfer, IVF-ET” and “chromosomal abnormalities or cytogenetic analysis” and “first trimester miscarriages or spontaneous abortion or pregnancy loss ” only permitted the articles published in English. The last search was updated on April 01, 2013. And then reference lists of the relevant studies were also examined and the literature retrieval was completed independently by two reviewers (Lihong Pang and Junzhen Qin). Discordance was settled by consultation of a third reviewer (Mujun Li). When a study reported the outcomes on different subtypes of ART, we considered it as separate studies in the meta-analysis.

### Eligibility of relevant studies

**Figure 1:**
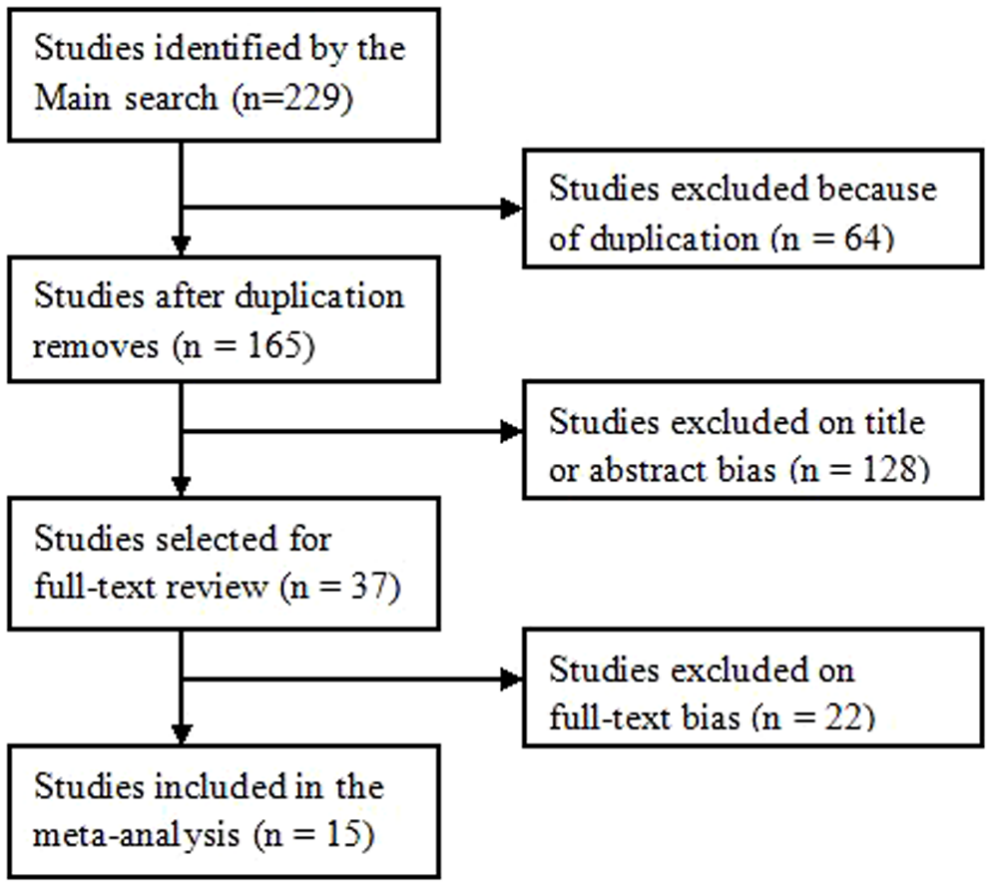
Flow diagram of included studies for this meta-analysis.

The main searching strategy identified 229publications, 64 publications were excluded because of duplication (Figure 1). Manual search of references cited in the published studies did not reveal any additional articles. We selected eligibility studies through reviewing abstracts of the remaining 165 articles and all citations. Published studies were included based on the following criteria: (1) case-control design; (2) evaluating the risk of chromosomal abnormalities occurred in first trimester miscarriages after ART; (3) the rate of abnormal chromosomes in first trimester miscarriages must offer in order to help us infer the results to estimate the odds ratio (ORs) and their 95% confidence intervals (CIs); and (4) studies published in English.

### Data extraction

Data extraction was conducted independently by two reviewers (Li-Hong Pang and Jun-Zhen Qin) from all included studies. Information was summarized as follow: the first author's name, publication date, study period, matching criteria, pregnancy loss definition, source of controls, the way of POC received, cytogenetic analysis obtained, method of conception, cytogenetic analysis and ratio of cases to control. The two reviewers examined the data extraction results and reached an agreement on all of the data extracted. If inconsistent opinion appeared on the data extraction results, a third reviewer (Xing Zhou) was invited to take part in discussion till the problem solved. Try best to communicate with the authors when data incomplete. Major characteristics are summed up in Table 1.

**Table 1:** Main characteristics of included studies.

Author	publication date	study period	matching criteria	pregnancy loss definition	source of control	way of POC received	cytogenetic analysis obtained	case/control	Conception method of case group	Conception method of control group	Quality scores
Plachot	1989	not stated	age	not stated	HB	not stated	not stated	34/30	ATR	NP	4
Sonntag	2001	not stated	age.ovatian stimulation	not stated	HB	not stated	peripheral lymphocytes using standard techniques	20/20	ATR/ICSI	NP	6
Causio	2002	1994– 1999	age.ovatian stimulation	not stated	HB	D&C	G-banded chromosomes peripheral blood lymphocytes or CV	79/63	IVF	ICSI	6
Tan	2004	2000–2002	age.ovatian stimulation	not stated	HB	D&C	Comparative genomic hybridizationCGH analysis of CV	33/8	IVF	ICSI	6
Lathi	2004	1999–2002	age	ultrasound evaluation alone or combination with serum hCG monitoring.	HB	D&C	CGH or cytogenetic analysis of CV	38/21	IVF	ICSI	7
Ma	2006	1999–2003	not stated	not stated	HB	D&C	CGH or cytogenetic analysis of CV	34/56	IVF	ICSI	4
Massie	2008	1999–2006	age,reproductive								
history	not stated	HB	D&C	cytogenetic analysis of CV	131/30	ART(IVF+ICSI)	NP	6			
Bettio	2008	2002–2005	age	not stated	HB	D&C	cytogenetic analysis of CV	133/144	ART(IVF+ICSI)	NP	6
Kushnir	2009	2000–2006	age	not stated	HB	D&C	cytogenetic analysis of CV	159/196	IVF	ICSI	6
Martinez	2010	1996–2007	age	not stated	HB	D&C	cytogenetic analysis of CV	451/136	ART(IVF+ICSI)	NP	6
Kim	2010	2005– 2009	age,Previous pregnancy status	not stated	HB	D&C	cytogenetic analysis of CV	254/128	ART(IVF+ICSI)	NP	5
Kroon	2011	2007–2009	age	ultrasound evaluation alone or combination with serum hCG monitoring.	HB	D&C	CGH or cytogenetic analysis of CV	118/234	ART	NP	7
Bingol	2012	not stated	age.ovatian stimulation	not stated	HB	D&C	CGH or cytogenetic analysis of CV	71/81	ICSI	NP	4
Werner	2012	2001–2010	age	absence of fetal cardiac activity	HB	D&C	CGH or cytogenetic analysis of CV	276/23	ART(IVF+ICSI)	NP	6
Li	2012	2011–2012	age,BMI	not stated	HB	D&C	SNP arrays CV	65/16	ATR	NP	6

HB, hospital-based; D&C, dilatation and curettage; CV, chorionic villi; POC, products of conception; NP, natural pregnancy.

Quality of the studies was determined with the use of the partially validated Newcastle-Ottawa Quality Assessment Scale: Case-Control Studies (NOS; http://www.Iri.ca/programs/ceu/oxford.htm). The total scores ranged from 0 (lowest) to 9 (highest), and a study with scores≥6 was considered a high-quality study, whereas studies with scores<6 were classified as low-quality studies.

### Statistical analysis

Odds ratios (OR) with 95% confidence intervals (CI) for dichotomous data were used to assess the risk of chromosomal abnormalities in different compared models: ART versus natural pregnancy (NP); ICSI versus NP; IVF versus NP; IVF versus ICSI. The statistical heterogeneity among studies was detected by χ^2^ tests and I^2^ test. When the result of χ^2^ tests (a P value of <0.1) or I^2^>50%, indicating the existence of statistically significant heterogeneity, a random-effects model (the DerSimonian and Laird method) was used to combine the data [14]. If the P value ofχ^2^ tests >0.1 and I^2^<50%, showing the absence of heterogeneity, a fixed-effects (the Mantel–Haenszel method) was used [15]. For each compared model, Begg's funnel plot and Egger's regression asymmetry test [16] were used for evaluating publication bias. To explore the sources of heterogeneity among studies, we performed logistic meta-regression and subgroup analysis. The following study characteristics were included as covariates in the meta-regression analysis: cytogenetic analysis of chorionic villi (Yes versus no), pregnancy loss diagnosis (Yes versus no), and quality score (high quality studies versus low quality studies). Subgroup analyses were conducted by stratification of cytogenetic analysis of chorionic villi and pregnancy loss diagnosis. Galbraith plots analysis was performed for further exploration of the heterogeneity. Sensitivity analysis was performed by sequential omission of a single study for searching out the influence of each data included in the meta-analysis. All analyses were conducted using Stata/SE 12.0 for Windows (Stata Corp LP, College Station, USA). To guarantee the reliability and the accuracy of the results, two authors typed the data into the statistical software programs independently with the same results.

## Results

### Study Characteristics

According to the inclusion and exclusion criteria, 15 studies relevant to the risk of chromosomal abnormalities resulting in a first trimester miscarriage after ART were identified (Figure 1) [17]–[30]. Among them, six of the eligible studies contained data on two different subtypes of ART(ICSI and IVF), we considered it as separate studies. Therefore, a total of 10 separate comparisons for ART vs. NP, 7 separate comparisons for ICSI vs. NP, 6 separate comparisons for IVF vs. ICSI and 10 for IVF vs. ICSI finally included in our meta-analysis. Eight studies evaluated the risk of embryonic chromosomal abnormalities in advance maternal age. Therefore, a total of 10 studies including 1,521 cases and 1,116 controls were available for the meta-analysis of ART vs. NP, 7 studies containing 602 cases and 562 controls were included for ICSI vs. NP, 6 studies containing 539 cases and 491 controls were included for ICSI vs. NP, 10 studies containing 848 cases and 859 controls were included for IVF vs. ICSI and 8 studies containing 601 cases and 602 controls were included for maternal age≥35 vs. <35. The main characteristics of the studies were summarized in Table 2. Of all the eligible studies, 13 were conducted in cytogenetic analysis of chorionic villi (CV) by dilation and curettage, and 2 were not; 3 were given the definition of pregnancy loss. And the other 12 were not. The source of controls in all the eligible studies included was hospital–based.

**Table 2:** Frequency of abnormal karyotypes in eligible studies.

study	year	Trisomy	monosomy X	Triploidy	Tetraploidy	Double trisomies	Structural anomalies	Autosomal monosomy	Masaic	47.XXY	Polyploidy
Plachot	1989	66.67%	14.29%	4.76%	4.76%	4.76%	4.76%				
Causio	2002	62.07%	24.14%	6.90%	6.90%						
Lathi	2004	84.38%	3.13%	6.25%			6.25%				
Ma	2006	58.00%	10.00%		4.00%	14.00%	14.00%				
Bettio	2008	71.35%	7.57%	8.11%	3.78%	5.95%	3.24%				
Massie	2008	86.91%	3.74%	9.35%							
Kushnir	2009	78.09%	3.37%							6.74%	
Kim	2010	72.94%	6.88%	6.88%		6.42%					6.88%
Martinez	2010	59.47%	8.28%			5.62%	5.62%	0.59%	8.58%	0.30%	11.54%
Kroon	2011	68.11%	8.70%	11.59%	1.45%		5.07%		5.07%		
Bingol	2012	56.00%	17.33%	17.33%						6.67%	
Werner	2012	90.91%	4.55%				4.55%				
Li	2012	62.50%	3.57%	5.36%			21.43%	1.79%	5.36%		

A total of 3278 patients with first trimester abortions were included in our analysis cytogenetic analyses. Cytogenetic analysis of products of conception (POC) indicated that 1603(48.9%) were karyotypic abnormalities, and among them 1143 (71.3%) were autosomal trisomy, 118 (7.3%) were monosomy X, 62(3.8%) were structural anomalies, 54 (3.37%) were polyploidy, 39 (2.4%) were mosaic, 33 (2.1%) were double trisomies, and14 (0.87%) were tetraploidy. Therefore, autosomal trisomies represented the most common chromosomal abnormalities in spontaneous abortions from our data. Table 2 shows the details of the cytogenetically abnormal conceptuses.

### Meta-analysis results

**Figure 2:**
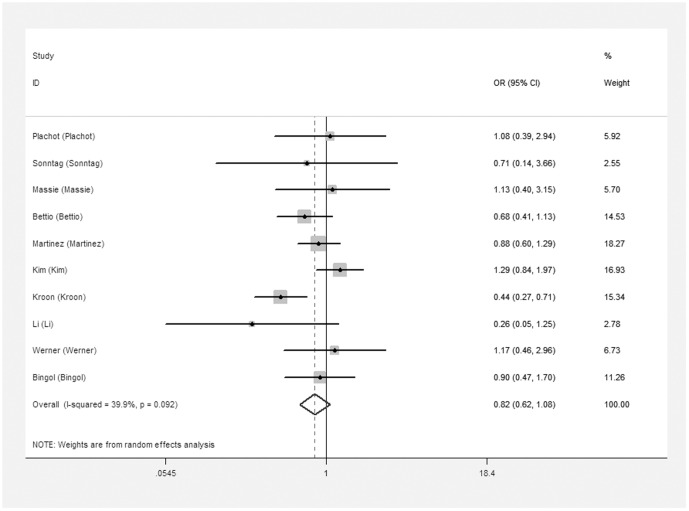
Forest plots for risk of chromosomal abnormalities in ART compared with NP.

**Figure 3:**
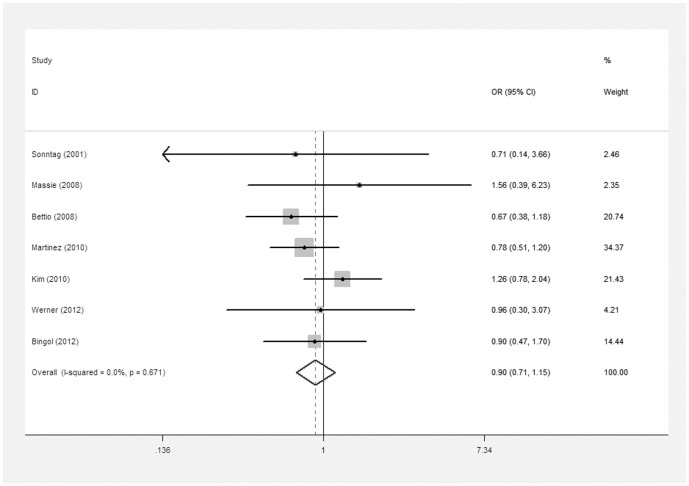
Forest plots for risk of chromosomal abnormalities in ICSI compared with NP.

**Figure 4:**
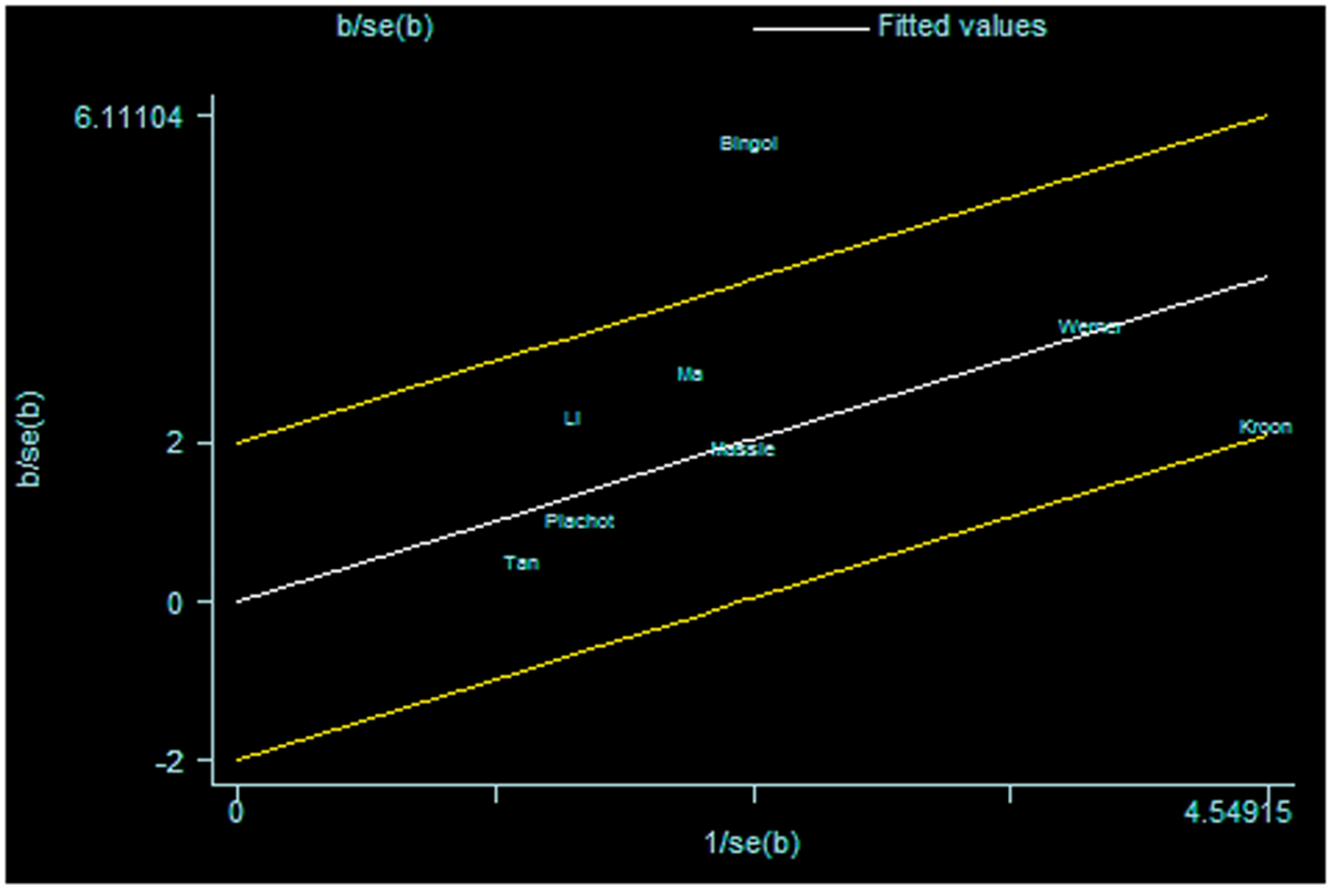
Forest plots for risk of chromosomal abnormalities in IVF compared with NP.

**Figure 5:**
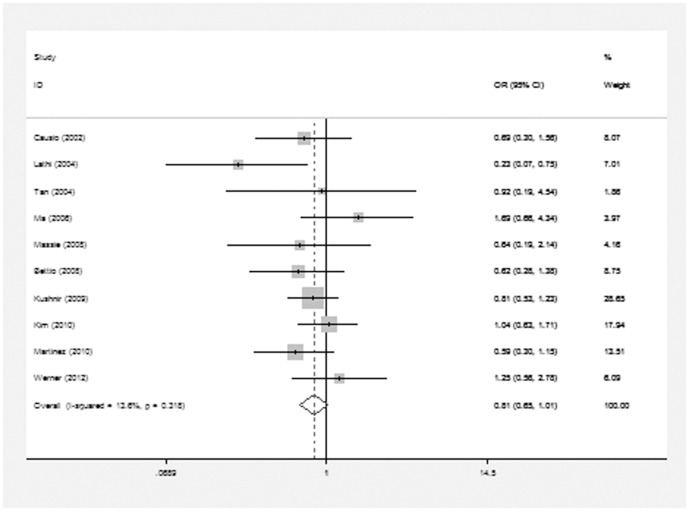
Forest plots for risk of chromosomal abnormalities in IVF compared with ICSI.

The meta-analysis of the compared model for ART vs. natural pregnancy indicated that risk of chromosomal abnormalities resulting in a first trimester miscarriage after ART was not significantly increased (10 studies, random effects OR 0.82, 95% CI: 0.62–1.08, heterogeneity χ^2^: P = 0.092, I^2^ = 39.9%; Figure 2]. In addition, we failed to identify any significant association between the different types of ART using and the risk of chromosomal abnormalities occurring in a first trimester miscarriage. For ICSI vs. natural pregnancy, insignificant elevated risk of embryonic chromosomal abnormalities was detected (7 studies, fixed effects OR 0.90, 95% CI: 071–1.15, heterogeneity χ^2^: P = 0.67, I^2^ = 0.0%; Figure 3). For IVF vs. natural pregnancy, only 6 studies included in the meta-analysis (6 studies, fixed effects OR 0.87, 95% CI: 0.64–1.18, heterogeneity χ^2^: P = 0.125, I^2^ = 42.1%; Figure 4). For IVF vs. ICSI, no significantly different risk was yet found (10 studies, fixed effects OR 0.81, 95% CI: 0.65–1.01, heterogeneity χ^2^: P = 0.318, I^2^ = 13.6%; Figure 5).

**Figure 6:**
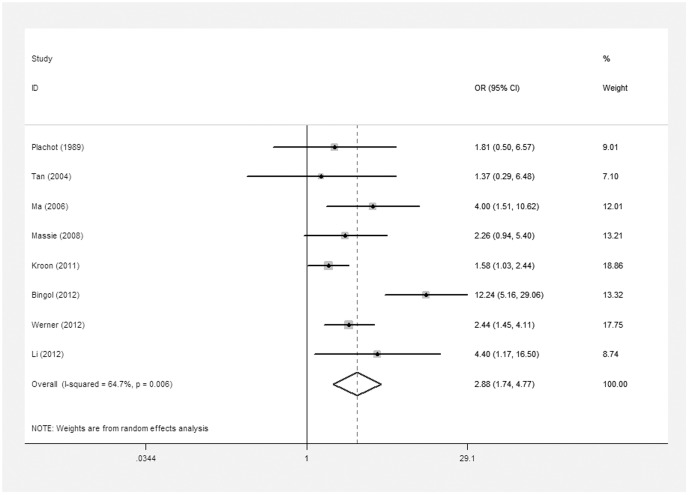
Forest plots for risk of chromosomal abnormalities in maternal age≥35 compared with <35.

In order to explore the relationship between advance maternal age and risk of embryonic chromosomal abnormalities, maternal age was subdivided into the following groups: <35 years and 35≥ years in this meta-analysis. We observed significant increase rate in embryonic chromosomal abnormalities with advance maternal age (8 studies, random effects OR 2.88, 95% CI: 1.74–4.77, heterogeneity χ^2^: P = 0.006, I^2^ = 64.7%; Figure 6).

### Heterogeneity Analysis

For the compared models of ART versus natural pregnancy and maternal age≥35 versus<35, the I^2^ values of heterogeneity were greater than 50% and the P values were lower than 0.10, which indicated statistically significant heterogeneity between studies. We performed meta-regression to explore the sources of heterogeneity. Meta-regression analysis of data did not show that the cytogenetic analysis of chorionic villi (Yes versus no), pregnancy loss diagnosis (Yes versus no), or quality score (high quality versus low quality) were the major sources which contributed to heterogeneity. The three covariates were failed to relate with the ORs in the compared model of ART vs. NC (regression coefficient = 0.852, 95%CI: 0.259–2.800, p = 0.765 for cytogenetic analysis of chorionic villi; regression coefficient = 0.623, 95%CI: 0.319–1.217, p = 0.142 for pregnancy loss diagnosis and regression coefficient = 0.624, 95%CI: 0.343–1.136, p = 0.107 for quality score, respectively), maternal age model≥35 versus<35 (regression coefficient = 1.676, 95%CI: 0.169–16.627, p = 0.602 for cytogenetic analysis of chorionic villi; regression coefficient = 0. 537, 95%CI: 0.157–1.835, p = 0.262 for pregnancy loss diagnosis and regression coefficient = 0.395, 95%CI: 0. 134–1.165, p = 0.080 for quality score, respectively). Subsequently, subgroup analyses by quality scores, cytogenetic analysis of chorionic villi and pregnancy loss diagnosis indicated that heterogeneity still existed in ART vs. NP and maternal age≥35 vs. <35 comparison models.

**Figure 7:**
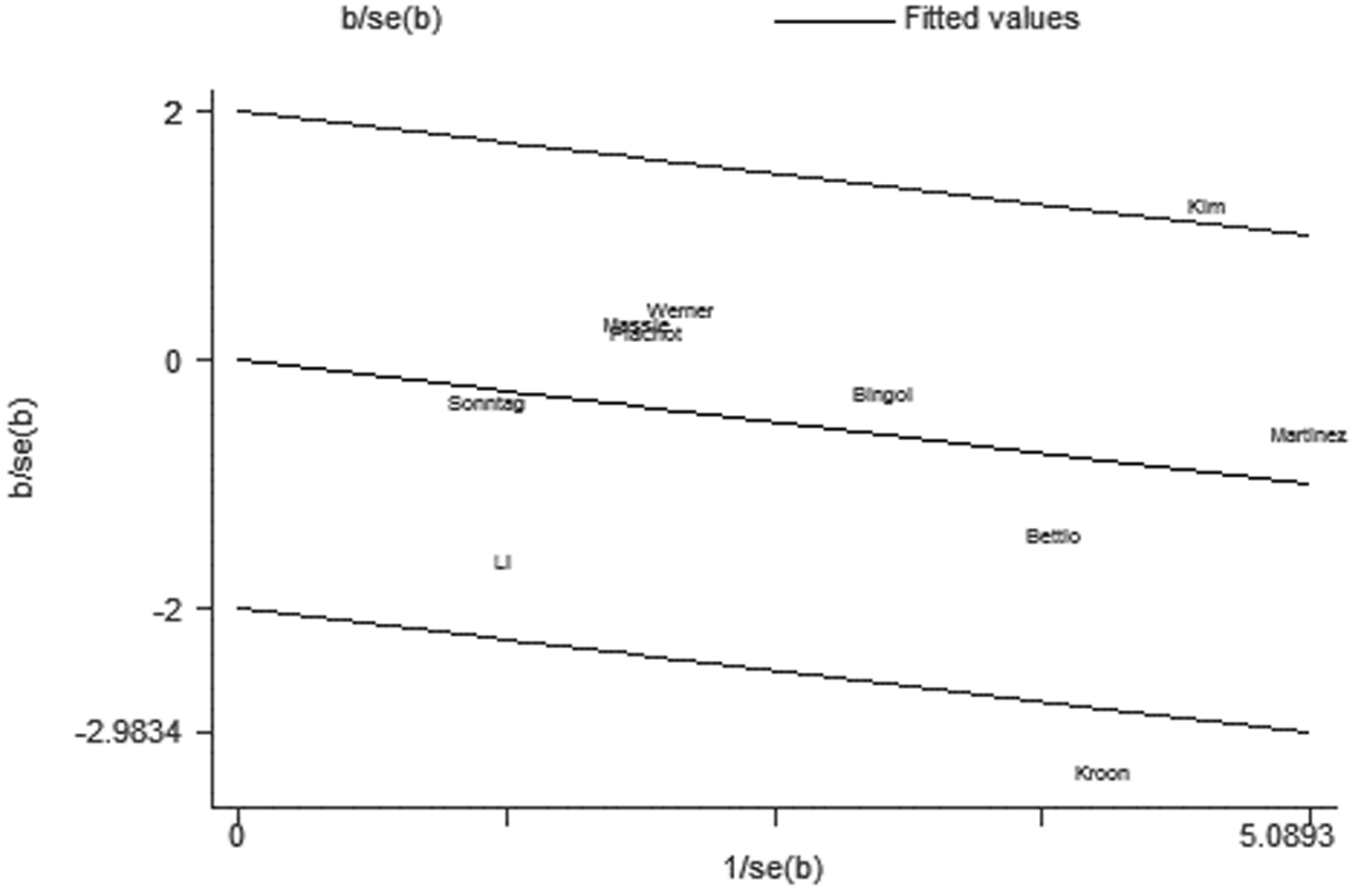
Galbraith plots for risk of chromosomal abnormalities in ART versus NP.

**Figure 8:**
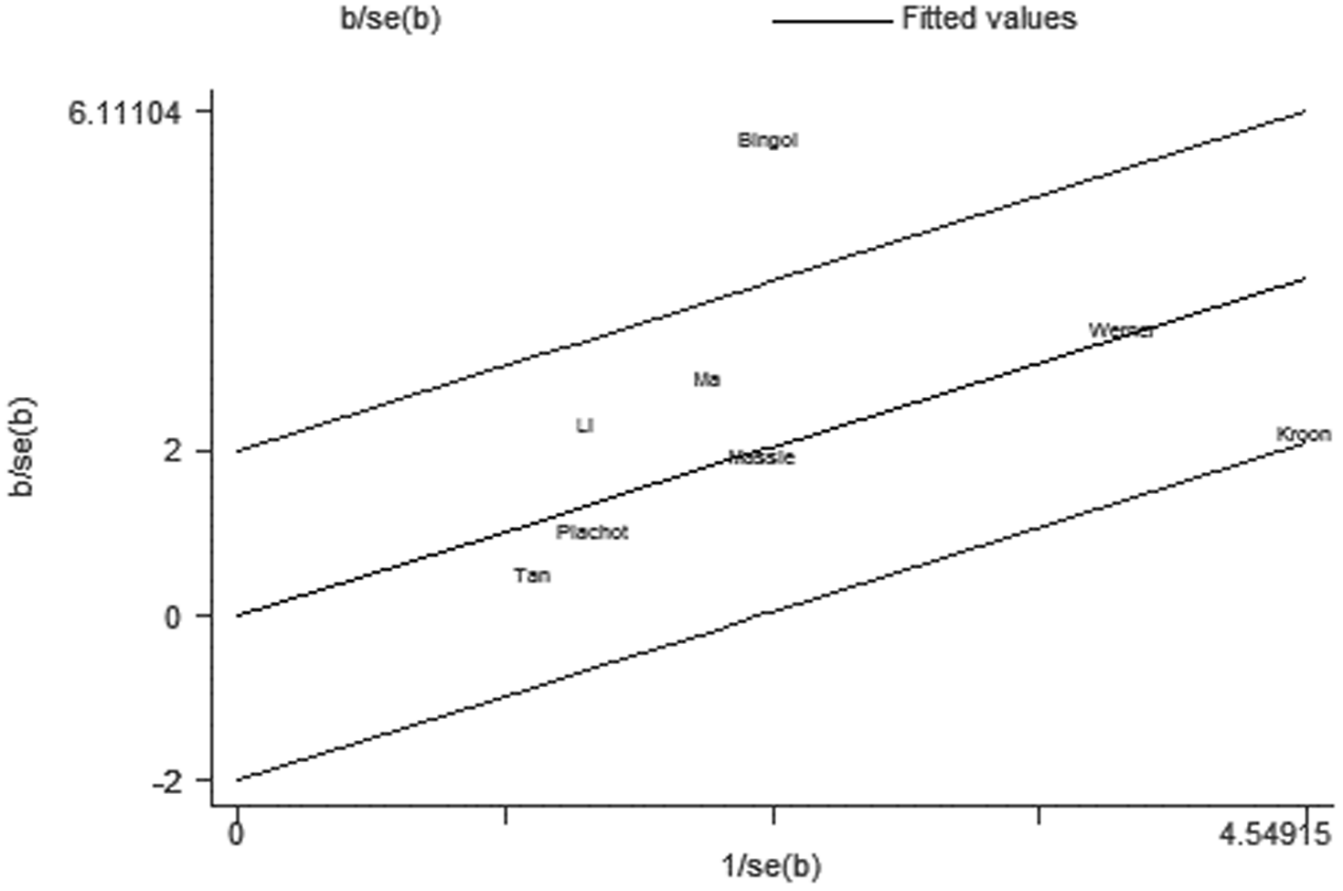
Galbraith plots for risk of chromosomal abnormalities in maternal age≥35 versus <35.

For further exploration of the heterogeneity we performed Galbraith plots analysis to identify the outliers which might contribute to the heterogeneity. Our results indicated that Kim et al. [26] was outliers in the compared model of ART vs. NC (Figure 7) and Bingol et al. [28] in the maternal age model (Figure 8). By excluding the outliers Kim et al. [26] and Bingol et al. [28] respectively in compared models of ART vs. NC and maternal age, I^2^ values decreased lower than 50% and P values were greater than 0.10 (ART vs. NC: P = 0.288, I^2^ = 17.4%;maternal age≥35 versus<35: P = 0.515, I^2^ = 0.0%). The summary ORs for compared models of ART vs. NC changed into significance after omitting the study of Kim et al. (fixed effects OR 0.73 (95% CI: 0.59–0.90), but the significance of summary OR for maternal age model was not influenced by excluding the study of Bingol et al.

### Sensitivity Analysis and Publication Bias

**Figure 9:**
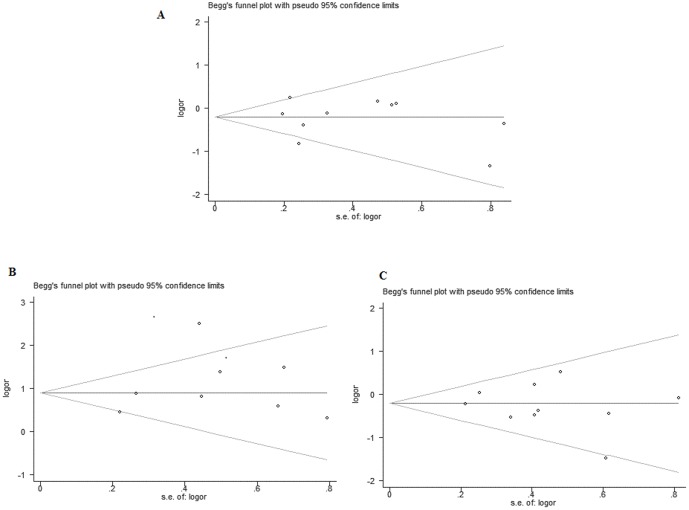
Funnel plot analysis to detect publication bias: A Funnel plot for compared model of ART vs.NP; B Funnel plot for maternal age≥35 vs. <35; C Funnel plot for IVF vs. ICSI.

To prove reliability of the available evidence, sensitivity analyses were performed to examine the influence of the individual data-set to the pooled ORs by sequentially omitting a single study each time. But in our results, the corresponding pooled ORs of chromosomal abnormalities rate in a first trimester miscarriage were not materially altered (data not shown), demonstrating that our results were statistically robust. Begg's funnel plot and Egger's test were performed to assess the publication bias of literatures in all comparison models. The shapes of the funnel plots did not suggest any obvious asymmetry (Figure 9). Then, the Egger's test was conducted to provide statistical evidence of funnel plot symmetry. The results still did not reveal any evidence of publication bias (P = 0.722 for ART vs. NC; P = 0.830 for IVF vs. NC; P = 0.548 for ICSI vs. NC; P = 0.970 for IVF vs. ICSI; P = 0.477 for the maternal age≥35 versus<35).

## Discussion

First trimester miscarriage is the most common complication of pregnancies conceived through assisted reproductive treatment (ART), with embryonic chromosome anomalies accounting for approximately 50% of these losses [6]. It is reported that the techniques employed for ART may have an increased risk of chromosomally abnormal products of conceptions compared to natural conception, and result in early pregnancy loss. Furthermore, it has been assumed that the risk of embryonic chromosomal abnormalities may be associated with different type of assisted reproductive technologies utilized [26]. This hypothesis was confirmed by our meta-analysis.

Our meta-analysis results indicated that infertile couples with ART treatment did not have an increased risk of chromosomal abnormalities resulting in a first trimester miscarriage compared to those with natural conception, especially among the different types of ART using (ICSI versus natural pregnancy; IVF versus natural pregnancy; IVF versus ICSI). However, when we excluded the study of Kim et al. [26] which was considered as an outlier of Galbraith plots analysis, a statistically significant increased chromosomal abnormalities risk was also found in the compared models of ART versus NP. In addition, significant increase rate was detected in embryonic chromosomal abnormalities with advance maternal age≥35 compared to <35. Actually, there are adverse factors of multiple ART failure, repeated miscarriages, advanced maternal age, altered karyotype, spermatozoa obtained by Mesa-Tese reported, which lead to a elevated risk of embryonic chromosomal abnormalities [9], [10]. The reasons why the techniques employed for ART did not indicate any increased risk of chromosomally abnormal miscarriages may included: the gametes selected in vitro in couples undergoing ART were high-quality, which would reduce the incidence of embryonic chromosomally abnormal abortions; in addition, genetic chromosome abnormality would potentially lead to lower successful embryo implantation rates under natural selection mechanisms, suggesting bias on study results.

With respect to compared model of maternal age≥35 vs. <35, 8 studies were found in our meta-analysis. Several studies have shown that increasing miscarriage and aneuploidy rates with increasing maternal age, and the main cause of this risk is considered to be errors arising at meisois I in the oocyte [31], [32], [33]. The theory of impact of chronologic ovarian aging has been provided to support the risk on fetal. Incidence of meiotic error in oocytes are elevated in women with advancing maternal age, which due to the prolonged time that oocytes spend arrested in meiosis I before ovulation [34], [35]. Additionally, meiotic spindle morphology has been found to transform deleteriously with increasing maternal age [36].

Heterogeneity analysis of different compared models suggested significant heterogeneity in ART vs. NP and maternal age≥35 vs. <35. To explore the sources of heterogeneity, we performed meta-regression and subgroup analyses. Meta-regression analysis of maternal age data showed that the quality scores but not cytogenetic analysis of chorionic villi (Yes versus no), pregnancy loss diagnosis (Yes versus no) might substantially influence the initial heterogeneity, whereas this three covariates were failed to explain the significant heterogeneity in the compared model of ART vs. NC. Subgroup analyses by quality scores, cytogenetic analysis of chorionic villi and pregnancy loss diagnosis indicated that heterogeneity still existed in ART vs. NP and maternal age≥35 vs. <35 comparison models. To further investigate the heterogeneity, Galbraith plots analysis was performed to identify the outliers which might contribute most to the heterogeneity. Our results indicated that Kim et al. [9] was outliers in the compared model of ART vs. NC and Bingol et al. [18] in the maternal age model. All I^2^ values decreased lower than 50% and P values were greater than 0.10 after excluding the studies of Kim et al. [9] and Bingol et al. [18] respectively in ART vs. NP and maternal age≥35 vs. <35 comparison models. The summary ORs for compared models of ART vs. NC changed into significance after omitting the study of Kim et al. (fixed effects OR 0.73, 95% CI: 0.59–0.90). The results indicated that the study of Kim et al. [18] was the main source of heterogeneity for the risk of chromosomal abnormalities in ART vs. NC. In addition, by excluding the study of Bingol et al. the significance of summary OR for maternal age model was not were not material altered, which demonstrated that our data were robust and reliable. The results indicated that the study of Bingol et al. might be the major source of the heterogeneity for the maternal age≥35 vs. <35. Caution is always required when checking the efficacy of these attempts.

It should be noted that the present meta-analysis existed some limitations, caution is always required when interpreting the results. First, all of the data included in our meta-analysis were not based on individual adjusted ORs, individuals of some studies were not matched by maternal age and reproductive history within the case and control groups. To guarantee synthesis of the best available evidence, a more accurate evaluation should be adjusted by potentially suspicious factors, including maternal age, reproductive history, the number of embryos transferred and spermatozoa obtained. Second, the methods of ovarian stimulation were not uniformly defined in our analysis. Therefore, whether this confounding factor of ovarian stimulation may play a role on the risk of chromosomally abnormal miscarriages wan unclear. Third, the number of studies included in this study for compared models of ICSI vs. NP and IVF vs. NP were small. Forth, published bias may result from the unpublished data, and languages of the articles included in our meta-analysis only permitted published in English.

In conclusion, the present meta-analyses t suggested infertile couples with ART treatment did not have an increased risk of chromosomal abnormalities occurring in a first trimester miscarriage compared to natural conception, and no statistical difference in frequency of chromosome anomaly among the different types of ART utilized, whereas the risk of fetal aneuploidy significantly increased with maternal age≥35. Although spontaneous abortion of ART may be a natural selection way of optimization to reduce the occurrence of birth defects, it not only decreases the successful rate of ART but also adds emotional pressure as well as economic burden to the infertile couples. Therefore, large sample studies with standardized unbiased ovarian stimulation methods, the number of embryos transferred, spermatozoa obtained and well matched controls for further investigation should be conducted. Such knowledge may help better preventing the occurrence of early spontaneous abortion, taking intervention measures to birth defects on time, and greatly increasing the take home baby rate.

## Supporting Information

Checklist S1 **PRISMA checklist.**
(DOC)
